# Quantifying lactulose and mannitol using LC-MS/MS in a clinical study of children with environmental enteric disease

**DOI:** 10.1590/1414-431X2024e14156

**Published:** 2025-03-03

**Authors:** L.M.V.C. Magalhães, F.A.P. Rodrigues, J.Q. Filho, R.N.D.G. Gondim, S.A. Ribeiro, T.B.M. Rôla, M.A.F. Clementino, B.L.L. Maciel, P.J.C. Magalhães, A. Havt, A.A. Santos, A.A.M. Lima

**Affiliations:** 1 Centro de Biomedicina, Universidade Federal do Ceará, Fortaleza, CE, Brasil; 2 Departamento de Fisiologia e Farmacologia, Faculdade de Medicina, Universidade Federal do Ceará, Fortaleza, CE, Brasil; 3 Departamento de Educação Física e Esporte, Instituto Federal de Educação, Ciência e Tecnologia do Ceará, Fortaleza, CE, Brasil; 4 Faculdade de Medicina, Centro Universitário Christus, Fortaleza, CE, Brasil; 5 Programa de Pós-Graduação em Nutrição, Departamento de Nutrição, Universidade Federal de Rio Grande do Norte, Natal, RN, Brasil

**Keywords:** Environmental enteric disease, Intestinal epithelium barrier function, Lactulose mannitol permeability test, Intestinal epithelium dysfunction, Liquid chromatography coupled with tandem mass spectrum

## Abstract

Dysfunction of the intestinal epithelium barrier (DIEB) is frequent and can lead to serious complications in early childhood when diagnosis and clinical intervention are limited, especially in children with environmental enteric disease and malnutrition. The use of refined analytical techniques is increasingly necessary in this context. This study aimed to validate the high-performance liquid chromatography method coupled with tandem mass spectrometry (LC-MS/MS) to measure DIEB by lactulose:mannitol ratio detection (LM test) in samples of children with different social profiles from Fortaleza, Ceará. The first experimental set was conducted to validate the method through laboratory parameters, such as limit of detection (LD), limit of quantification (LQ), specificity/selectivity, linearity, accuracy, and precision. All validation parameters achieved detection and recovery standards within an acceptable coefficient of variation. Community samples (human development index (HDI) from 0.000 to ≤0.499) were obtained from children from the cohort study Malnutrition-Enteric Diseases, Fortaleza-CE (environmental enteric disease; EED group). The control group samples came from a school located in a region with a high HDI (>0.8). Mannitol excretion was lower in the EED group than in the control group (P<0.0001). On the other hand, LM was higher in this group compared to the control group (P<0.0001). For the first time, a robust analytical approach was used to detect biomarkers of environmental enteropathy (LM) in community samples, confirming with high-sensitivity the damage to the intestinal epithelial barrier function in populations living in low socio-economic conditions.

## Introduction

Intestinal epithelium dysfunction is a condition associated with environmental enteric disease (EED), which is characterized by malabsorption and diarrhea and results in irreversible deficits in physical and intellectual growth (1). EED associated with dysfunction of the intestinal epithelium barrier (DIEB) may trigger growth delay in young children (2). Stunting (length/height-for-age z-score <-1) is a widespread problem in low- and middle-income countries (LMICs) around the world, being associated with increased mortality, neurocognitive deficit, and low responses to oral vaccines (1,2).

DIEB with EED manifests as an acute and chronic condition of the small intestine that combines loss of villous architecture, reduced absorptive function, and intestinal and systemic inflammation with considerable limitation of morphological identification (3,4). In this context, recent studies have highlighted the need to develop refined techniques to measure intestinal epithelial barrier disorders (4,5).

The need to demonstrate the quality of chemical complexities in a clinical context through their comparability, traceability, and reliability is increasingly recognized and applied. Unreliable analytical data can lead to disastrous decisions and irreparable financial losses. In recent years, the use of analytical platforms in the clinical context has been increasingly recommended due to their broad applicability across various scenarios. Methods based on mass spectrometry are particularly suited for this purpose, given their exceptional capacity for detecting biomarkers associated with cellular functions. These methods enhance diagnostic accuracy through efficient identification, high precision, and specificity, which can be used to update current clinical protocols and develop new approaches (6-8). To ensure that any new analytical method provides reliable and interpretable results, it must undergo a validation process (9). Liquid chromatography-tandem mass spectrometry (LC-MS/MS) is a promising technique for testing with sugars to identify morphological and functional disorders of the intestinal epithelium, one of the most challenging clinical conditions to diagnose.

The lactulose:mannitol test (LM test) is a quantitative assay that measures two sugar molecules permeating across the functional intestinal epithelium barrier (10). It consists of the oral administration of a solution containing lactulose and mannitol, followed by the collection of urine for a period of 5 h. Both sugars are absorbed through permeation of the intestinal epithelium barrier, are not metabolized in the body, and are excreted in the urine through the process of glomerular filtration. Due to its low molecular weight (mw: 182 Da) and hydrophilic characteristics, the monosaccharide mannitol passes through the intestinal epithelium through hydrophilic pores via the transcellular route. The disaccharide lactulose (mw: 342 Da) has a higher molecular weight and passes into the normal intestine in low amounts via the paracellular route. The reduction in villus length with consequent reduction in the absorption area reduces the absorption of mannitol and the permeation of lactulose. On the other hand, the increase in intercellular space permeation or damage to the intestinal epithelium barrier function results in increased permeation of lactulose. Thus, the lactulose:mannitol ratio is associated with changes in the absorption area, permeability, and damage to the barrier function of the intestinal epithelium.

Due to the difficulty of applying reliable and validated methods to identify DIEB in needy children, we initially present the development of a high-performance liquid chromatography method coupled with tandem mass spectrometry (LC-MS/MS) to measure mannitol and lactulose sugars. Then, we evaluate the intestinal barrier function in children with EED compared to healthy control children living in the city of Fortaleza, Ceará, Brazil.

## Material and Methods

### Standard and calibration solutions

Standard solutions of lactulose, mannitol, and sorbitol were prepared by dissolving them in eluent B (see below), with acetonitrile and formic acid (0.05%) as the mobile phase. Individual stock solutions of each analyte were prepared at a concentration of 20 µg/mL and then were diluted in eluent B to 10, 50, 100, 500, 1000, 1500, and 2000 ng/mL. The final concentration of the internal standard (sorbitol) was 100 ng/mL.

### HPLC-MS/MS platform for validation and measurement of lactulose and mannitol levels

The LC-MS/MS system consisted of a high-performance liquid chromatography instrument (Agilent, USA) with a 1200 series LC pump, degasser, autosampler, and column oven coupled to a Q-TRAP 5500 triple quadrupole mass spectrometer with an electrospray ionization interface (ESI) (ABSciex, USA). For the separation of the compounds by LC, a HILIC-ZIC^®^ analytical column (ES Industries, USA) was used. MS/MS parameters for monitored ion transitions were obtained using 10 ng/mL solutions of each substance at a flow rate of 10 µL/min in the negative ionization mode.

The mobile phase for chromatographic separation was composed of an aqueous fraction (eluent A) and an organic fraction (eluent B). The aqueous fraction contained a mixture of a 5.0 mmol/L ammonium acetate solution, and the organic fraction contained acetonitrile acidified with 0.05% formic acid. The mobile phase composition was altered for gradient elution based on the study by Kubica et al. (11) (Supplementary Table S1). The parameters used for analytical validation were specificity, selectivity, linearity, limit of detection (LD), limit of quantification (LQ), accuracy, precision (repeatability and intermediate precision), and matrix effect.

### Preparation of standard solutions and urine samples

Each 500 µL urine sample was initially diluted in 500 µL of ultrapure water, and 100 mg of Amberlite MB150 ion exchange resin (Sigma-Aldrich, USA) was added to the diluted samples to eliminate excess sodium ions (12). The samples were vortexed for 3 min and then centrifuged for 6 min at 9961 *g* and 6°C. Thereafter, 10 μL of the supernatant was added to 100 μL of the internal standard sorbitol solution (100 ng/mL) and diluted again in eluent B to a total volume of 2 mL. Lactulose, mannitol, and sorbitol were dissolved in four urine samples free of compounds of interest at a concentration of 20 µg/mL each. The enriched samples were prepared as previously described (13). The final concentrations of the samples were 1000, 1500, 3000, and 6000 ng/mL for each substance. The prepared spiked samples were analyzed using LC-MS/MS. The calculation of LD and LQ was based on the standard deviation of the sample readings at a concentration of 100 ng/mL and the slope of the calibration curve across a range of concentrations (10, 50, 100, 500, 1000, 1500, and 2000 ng/mL). According to the equations used, we calculated LD as 3 times the standard deviation (SD) of the sample readings at 100 ng/mL. Similarly, the LQ was calculated as 10 times the SD (14).

### Preparation and performance of the lactulose:mannitol test

After fasting for at least 5 h, the children ingested lactulose (5 g) and mannitol (1 g) diluted in 20 mL of water. Children weighing less than 10 kg ingested 2 mL/kg of this solution. We then determined the amount of lactulose and mannitol recovered from a urine sample taken in 5 h with 50 µL of 20 µg/mL chlorhexidine solution added per each 50 mL of urine as a preservative. Lactulose and mannitol analytes excreted in the urine samples were measured using LC-MS/MS.

### Study design, population, and ethical approval

To validate the LC-MS/MS method in clinical specimens, we used urine samples from intestinal permeability tests done in children. The clinical study has been performed on 46 children enrolled in the cohort study Malnutrition-Enteric Disease (MAL-ED) (15) from the Canarinho Elementary school at Fortaleza, Ceará, Brazil. As a control group, healthy children from other Elementary schools were recruited in Fortaleza. All children were examined with permission from the mother or primary caregiver responsible for legal custody of the child. The study protocol and consent form were approved by the Institutional Review Board of the Federal University of Ceará and the National Council for Ethics in Research (Approval number: 238/05). We included children aged 0.5-7 years. Children with EED were defined as those from the cohort study MAL-ED from an urban area with low human development index (HDI: 0.000 to ≤0.499) in Fortaleza (15) that has poor environmental and hygiene conditions and the population is under a high risk of enteric pathogen contamination associated with increased inflammatory biomarkers in stool samples. Healthy control children were from a high HDI (>0.80) region in Fortaleza with excellent socio-economic and hygiene conditions and low risk of enteric pathogen contamination. Exclusion criteria defined in the study protocol were children with a history of hospitalization or serious health issues, such as human immunodeficiency virus infection, tuberculosis, neonatal illness, kidney disease, or other illnesses diagnosed by a physician. In addition, children with a parent or a primary caregiver with cognitive deficits or younger than 16 years of age were excluded.

### Sample size and statistical analysis

The lactulose:mannitol urinary excretion ratio was selected as the primary outcome variable and was used to calculate the sample size for LM test validation. To detect a 30% change in the lactulose:mannitol ratio at P=0.05 and 80% power, a sample of 17 participants was required in each subgroup. This calculation was based on data from preliminary studies in the same population (lactulose:mannitol ratio=0.13±0.04) (10). Data were entered into spreadsheets (Microsoft Access software; Microsoft Corporation, USA) and verified by two independent researchers to ensure accuracy. All data were de-identified, and statistical analysis was performed using SPSS Statistics 20.0 (IBM, https://www.ibm.com, USA). For analytical processing and measurement of mannitol and lactulose biomarker levels using LC-MS/MS, we used Analyst 1.4.1 software (http://www.appliedbiosystems.com). We used the Shapiro-Wilk test to assess the normality of data distribution and Levene's test to assess the equality of variances. For nonparametric variables, we used the Mann-Whitney U test for independent samples. For qualitative variables, we used the chi-squared test or Fisher's exact test. We used analysis of covariance (ANCOVA) to explore the influence of age on the differences in the lactulose:mannitol ratio between healthy children and children with EED. The results were considered significant if P<0.05.

## Results

The precursor ions of the standard lactulose, mannitol, and sorbitol analytes identified using the automatic method of direct multiple reaction monitoring in the mass spectrum is shown in Table 1. The component ions of interest were detected in the negative ESI mode. The highest intensity product ions in the detector were 160,952, 112,798, and 112,912 m/z for lactulose, mannitol, and sorbitol, respectively (Supplementary Figure S1). The automatic parameters detected for precursor and product ions of the lactulose, mannitol, and sorbitol standards were determined by flow injection analysis (FIA) coupled with LC (without the addition of the chromatographic column) and MS/MS. Supplementary Table S1 shows the chromatographic conditions when using the HILIC-ZIC^®^ column and selected FIA and MS/MS parameters, as well as product ions best quantified and selected from the precursor ions of lactulose, mannitol, and sorbitol standards based on calibration curves and correlation coefficients.

**Table 1:** Mass spectrum monitoring and operational parameters of precursor ions and product ions of lactulose, mannitol, and sorbitol, through multiple reaction monitoring (MRM).

Analytes	Precursor ions (m/z)	Product ions (m/z)	DP (V)	EP (V)	CE (V)	CXP (V)	Intensity^a^ (cps)
Lactulose	341.016	160.952	-75	-10	-1	-11	499000
		184.939			-48	-3	420000
		58.947			-48	-9	324000
		100.89			-22	-9	301000
		73.008			-36	-9	281000
Mannitol	180.932	112.798	-60	-10	-10	-9	230000
		136.813			-16	-9	193000
		58.994			-26	-9	124000
		71.009			-26	-5	122000
		89.033			-18	-7	109000
Sorbitol	180.935	112.912	-75	-10	-26	-9	225000
		58.924			-16	-7	150000
		136.927			-26	-9	143000
		70.973			-26	-9	131000
		92.879			-24	-11	107000

DP: Decomposition potential (voltage applied to the orifice to avoid clustering of ions); EP: entry potential; CE: collision energy; CXP: collision cell output potential; V: volts (voltage measurement); ^a^intensity of fragment ions by MRM.

Table 2 shows the precursor and product ions of the lactulose, mannitol, and sorbitol standard analytes with their respective calibration curve equations, detection and quantification limits, and correlation coefficients. The linear parts of the standard curves were in the concentration range between 10 and 2000 ng/mL. The correlation coefficients of the linear equations obtained for the three sugars were greater than 0.99. The calculations of LOD and LOQ were based on the standard deviation of the sample at a concentration of 100 ng/mL (the lowest concentration at which the method used was accurate and precise for each analyte) and the slope of the calibration curve in the region between 10 and 2000 ng/mL. The accuracy of the analytical method determined from the recovery and coefficients of variation of the standard analytes is summarized in Supplementary Table S2.

**Table 2:** Linearity, limit of detection (LD), and limit of quantification (LQ) of the method in the LC-MS/MS system for analysis of the excretion of lactulose, mannitol, and sorbitol sugars.

Analytes (precursor ion/product ions; m/z unit)	Calibration curve equation	LD (ng/mL)	LQ (ng/mL)	R
Lactulose (341.016/58.947)	y = 1.15e^4^x + 1.33e^6^	0.0055	0.0168	0.991
Mannitol (180.932/71.009)	y = 4.46e^4^x + 6.42e^6^	0.0003	0.0010	0.993
Sorbitol (180.935/58.924)	y = 5.03e^4^x + 7.10e^6^	0.0031	0.0001	0.995

R: correlation coefficient.

**Figure 1:**
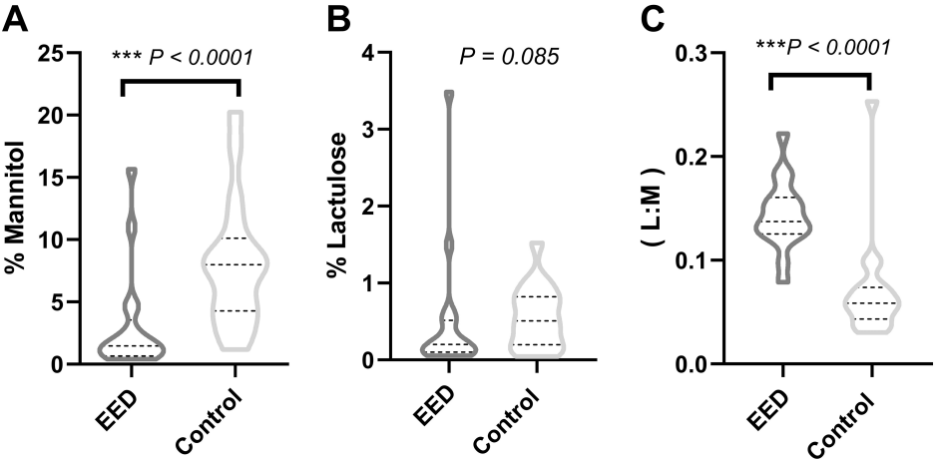
Distribution of mannitol (**A**) and lactulose (**B**) urinary excretion in healthy control and environmental enteric disease (EED) children. **C**, Lactulose:mannitol urinary excretion ratio. Mann-Whitney U test.

The L:M ratio was higher in the EED group than in the control children and the percent of mannitol excretion decreased in the EED group compared to the control children (Figure 1).

## Discussion

The application of an extremely refined analytical method for measuring biomarkers of the intestinal epithelium barrier function in the context of EED in the city of Fortaleza was evaluated for the first time. In the first experimental set, the tests aimed to standardize laboratory tests and obtain chemical parameters that could be used in the clinical context. Given the limitations of combining the separation of applied molecules and their possible detection into a single detection model, this was an innovative detection pattern. The method was then tested on samples of children in different social conditions who live in the same city, proving to be efficient for this purpose.

Several chromatographic methods have been used for the analysis of lactulose and mannitol in urine in EED and DIEB (16,17). However, the LC-MS/MS method proposed in this study for the analysis of lactulose and mannitol as biomarkers has advantages over liquid chromatography (LC) alone because of its higher sensitivity, specificity, and throughput (shorter sample run time) (10,11,16). However, this application occurs almost exclusively in study models, which are still a long way from hospital practice in Brazil.

To validate tests and exams for clinical settings, some parameters are required, such as linearity, reproducibility, and precision, among others. The linearity of the method was demonstrated by the addition of known amounts of standards (lactulose, mannitol, and sorbitol). The calibration curves for the working ranges of the three analytes showed satisfactory linearity (R≥0.991).

There are several approaches to assess accuracy and precision (repeatability and intermediate precision). Most commonly, the percentage of analyte recovery is calculated (17). The approach used here was designed to obtain the laboratory efficiency of the method using the LC-MS/MS technique. The coefficients of variation did not exceed 9.79%, which was below the limit suggested by Barboza Junior et al. (10). The recovery values obtained in order to assess the accuracy and repeatability of the method varied between 95.7 and 132.9, according to the concentrations analyzed. For intermediate precision, the recovery ranged between 100.94 and 139.76. Although recoveries close to 100% are desirable, the extent of analyte recovery up to 50-60% may be acceptable if the recovery is accurate and reproducible (17). The accuracy and precision parameters are key to the validation process. They are required for all method validation studies, except those with a qualitative purpose and intended only to demonstrate the presence of an analyte. For the quantitative analysis of trace elements, it is necessary to validate the detection and quantification limits. For the qualitative analysis, it is mandatory to validate only the detection limit. The evaluation of linearity is mandatory for new methods and qualitative analyses. The addition of a specific amount of ion-exchange resin to urine samples was sufficient to obtain high recovery values, as reported by Kubica et al. (11). For further details of the LC-MS/MS analytical method, please see Supplementary Tables S1 and S2.

Our laboratory procedures followed those of other analytical studies and obtained similar validation standards (11,18,19). Our findings were different from some of these studies due to the equipment used, the type of biofluid chosen for detection, the biomarker standards, and the chemical characteristics of each molecule (20-22). Several limitations are reported in studies when the aim is to determine sugars using LC. Some factors complicate detection, such as the type of biofluid to be analyzed, e.g. plasma, which makes it difficult to obtain in the context of EED, which is a silent condition that affects children in natural conditions and/or other clinical conditions (23,24).

The L:M urinary excretion ratio test has recently been considered one of the best noninvasive tests to assess the area of absorption, permeability, and damage to the intestinal epithelium barrier function (25). Increased lactulose is common in disorders of the intestinal epithelium associated with increased permeation of the paracellular pathway. On the other hand, mannitol permeation is associated with transcellular pathway. Both sugars are widely used to investigate these changes caused by enteric diseases (26,27). In this study, we developed and validated a new robust, sensitive, specific, and accurate HPLC-MS/MS method for measuring sugar biomarkers, such as lactulose and mannitol. The urinary lactulose excretion was similar in the groups of children with EED and in control healthy children. The LM permeability test was considered abnormal or positive for comparison purposes if the LM urine excretion ratio was >0.0864 (28). The LM ratio was higher in the EED group compared to the control group. Other previous studies have reported an increase in the LM ratio in children from underdeveloped countries in Africa and Asia (29,30).

Specifically, the rate of urinary mannitol excretion was significantly higher in the control group than in the EED group. It is well known that the decrease in mannitol permeation is associated with a reduction in the transport efficiency of micronutrients, ions, and solutes in general, both in clinical and pre-clinical models (31,32). In this way, it can be inferred that the EED children in the present study had absorptive impairment, consistent with the results of the noninvasive test to assess the urinary excretion rates of lactulose and mannitol. The method was shown to be applicable for the study of changes in the intestinal epithelium barrier function for follow-up and evaluation of preventive and curative interventions (33-35).

**Figure 2:**
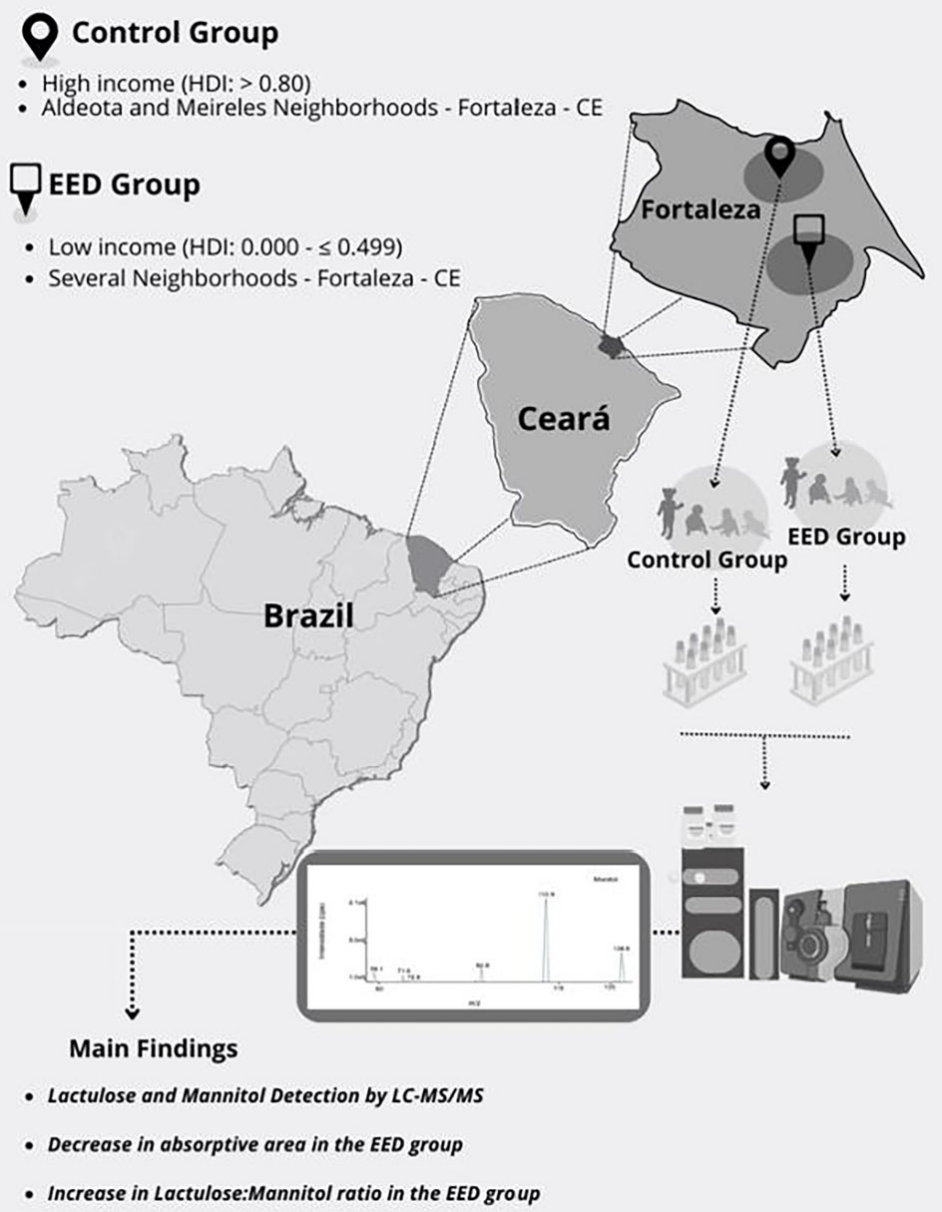
Overview of the methods and key findings from the experimental sets of the current study. LC-MS/MS: liquid chromatography method coupled with tandem mass spectrometry; EED: environmental enteric disease.

The advantage of this study was the use of a robust, highly sensitive, specific, and accurate method for determining lactulose and mannitol concentrations in urine. Although difficult to detect due to the proliferative nature and cellular self-renewal, the functional disorder was confirmed by our method. We speculate that environmental factors contributed to the dysregulation observed here (a summary of the findings is shown in Figure 2). Large cities like Fortaleza present numerous structural, social, and health inequalities that are typical to most metropolitan areas in the Northeast of Brazil (36). The lack of minimum economic conditions is associated with high manifestations of disease. These can manifest themselves in all age groups, but pose a critical risk when they affect early childhood (37).

Our study design and findings add a new diagnostic test to evaluate the tight junction permeability, one of most complex morphofunctional responses that lack investigative biomarker tools (38). Subtle differences in the intestinal epithelium barrier function were detected in children in early childhood from different socio-economic and hygiene conditions in the city of Fortaleza.

The limitation of the present method was the total urine collection time of 5 h, which makes collection difficult. Optimizing collection with reduced time could be an object of study in future work. Testing other molecules in EED children undergoing pharmacological and nutritional weight correction treatment could be an interesting object of study in disadvantaged developing areas.

In conclusion, the results of this study allowed us to propose LC-MS/MS as a robust, sensitive, specific, and accurate method for the determination of lactulose and mannitol to study the changes in intestinal epithelium barrier function in children with EED from low social-economic and hygiene conditions.

## Supplementary Material

Click to view [pdf].
