# Supplementary material for: Quantifying lactulose and mannitol using LC-MS/MS in a clinical study of children with environmental enteric disease
Source: Braz J Med Biol Res. 2025 Mar 3;58:e14156. doi: 10.1590/1414-431X2024e14156 (PMC11884778; doi:10.1590/1414-431X2024e14156)
Supplement: Supplementary file 1 [file 1414-431X-bjmbr-58-e14156-suppl.pdf]

Figure S1. Spectral and chromatographic analysis of mannitol, sorbitol, and lactulose using HPLC-MS/MS. **A**, On the X-axis, we have the mass-to-charge ratio (m/z) of the fragmented ions detected by the mass spectrometer. The m/z values indicate the specific ions generated during the analysis, highlighting the most relevant fragments. On the Y-axis, we can observe the relative intensity of the ions or signal abundance. The peak heights indicate the relative quantity of each detected ion (fragment). The most intense peak corresponds to the most abundant fragment. **B**, On the X-axis, we can observe the retention time, which is the time required for a compound to pass through the chromatographic column and be detected. On the Y-axis, we can observe the intensity, which is proportional to the quantity of the analyzed compounds.

A Representative spectrograms of the compounds mannitol, sorbitol and lactulose, showing the spectral characteristics of each substance

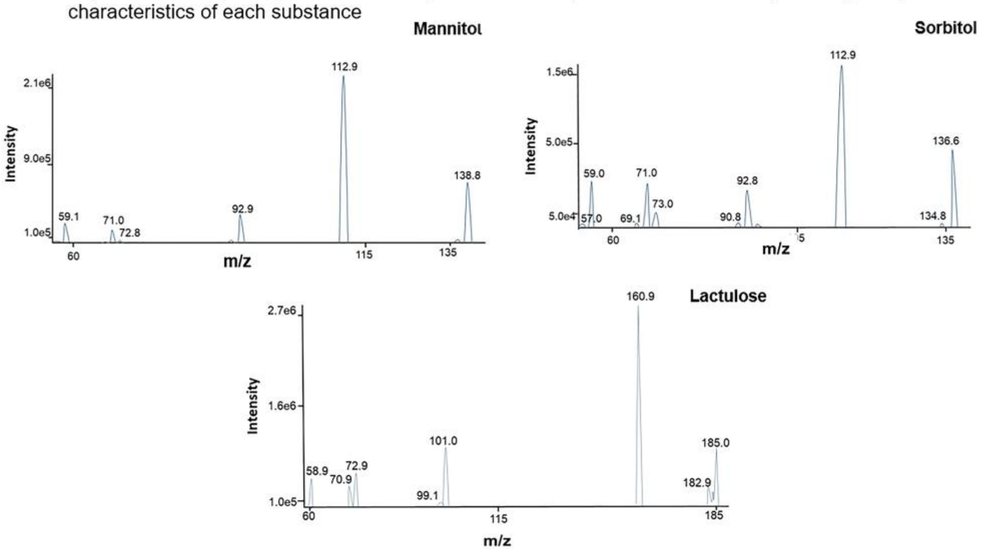

B Chromatogram of the compounds mannitol, sorbitol and lactulose, with specific retention times and the characteristic peaks associated with each analyte

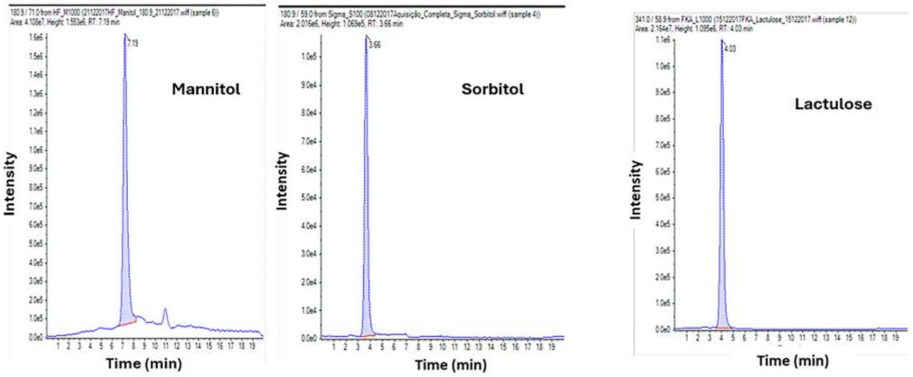

Table S1. Chromatographic column conditions (HILIC-ZIC®) and optimal parameters for monitoring transition ions.

Mobile phase gradient (%)	Eluent A (25/92/25) / Eluent B (75/10/75)
Flow	300 µL/min
Injection volume	20 µL
Column oven temperature	40°C
Analysis run time	10 min

Parameters for transition ions

Quantitative [Q][a] → qualitative [q][b] Precursor ion (m/z)[c] → product ion (m/z)		DP (V)	EP (V)	CE (V)	CXP (V)
Lactulose	^{Q}341.016 → 160.952^{q}	−135	−10	−12	−11
	^{Q}341.016 → 58.947^{q}			−48	−9
Mannitol	^{Q}180.932 → 112.798^{q}	−60	−10	−10	−9
	^{Q}180.932 → 71.009^{q}			−26	−5
Sorbitol	^{Q}180.935 → 112.912^{q}	−75	−10	−26	−9
	^{Q}180.935 → 58.924*			−16	−7

Mass spectrum operating parameters

		TEM (°C)	GS1 (psi)	CUR (psi)	GS2 (psi)	IS (V)	CAD
Lactulose	^{Q}341.016 → ^{q}160.952	500	50	20	40	–3500	Median High
	^{Q}341.016 → ^{q}58.947	500	50	20	45	–3500	
Mannitol	^{Q}180.932 → ^{q}112.798	450	50	20	50	–4000	Median High
	^{Q}180.932 → ^{q}71.009	550	50	22	50	–4000	
Sorbitol	^{Q}180.935 → ^{q}112.912	650	50	20	45	–4500	Median High
	^{Q}180.935 → ^{q}58.973*	450	40	30	40	–3500	
Selection of parameters for analysis		500	47	25	5	–3500	High

[a]Precursor ion detected by multiple reaction monitoring (MRM); [b]best quantitation product ion from the equation of the calibration curve and correlation coefficient. [c]mass/charge unit; DP: decomposition potential (voltage applied to the orifice to avoid clustering of ions; V); EP: entry potential; CE: collision energy; CXP: collision cell output potential; TEM: temperature at the source (Celsius); GS1: nebulizer gas (pound-force per square inch); CUR: gas flow between orifice and gas curtain; GS2: heating gas; IS: ion spray; CAD: gas collision; *ratio of precursor ion (m/z) to product ion (m/z) chosen to avoid coelution with mannitol.

Table S2. Repeatability of the method in the LC-MS/MS system to analyze the excretion of lactulose, mannitol, and sorbitol sugars.

Sugar	Initial concentration[a] (ng/mL)	Concentration obtained[b] (ng/mL) (n=3)	Recovery[c] (%)	SD	CV (%)
Lactulose (341.016/58.947)	100	95.7	95.7	1.71	1.8
	500	499.2	99.8	6.15	1.2
	1000	994.8	99.4	20.83	2.1
Mannitol (180.932/71.009)	100	112.9	112.9	11.06	9.8
	500	661.2	132.2	8.63	1.3
	1000	1116.4	111.6	18.71	1.7
Sorbitol (180.935/58.924)	100	90.4	90.4	2.25	2.5
	500	568.6	113.7	33.08	5.8
	1000	988.2	98.8	45.82	4.6

[a]Concentration in fortified samples; [b]concentration obtained through the average of the values obtained by the equation of the calibration curve of the spiked samples adding the standards in the samples of urine of volunteers; [c]percent recovery; SD: standard deviation; CV: coefficient of variation.
